# Calcaneal fracture maps and their determinants

**DOI:** 10.1186/s13018-022-02930-y

**Published:** 2022-01-21

**Authors:** Qingwen Yu, Zhirui Li, Jiantao Li, Qinghua Yu, Licheng Zhang, Daohong Liu, Mingzhu Zhang, Peifu Tang

**Affiliations:** 1 Medical School of Chinese People’s Liberation Army, No.28 Fuxing Rd, Haidian District, Beijing, 100853 People’s Republic of China; 2 Department of Orthopedics, Fourth Medical Centre of PLA General Hospital, Beijing, 100037 People’s Republic of China; 3 National Clinical Research Center for Orthopedics, Sports Medicine and Rehabilitation, Beijing, 100853 People’s Republic of China; 4 Department of Orthopedics, Chinese PLA General Hospital and Hainan Branch, Sanya, 572013 People’s Republic of China; 5 School of Basic Medicine, Air Force Medical University, Xi’an, Shanxi 710032 People’s Republic of China; 6 Center of Foot and Ankle Surgery, Beijing Tongren Hospital, Capital Medical University, 1 Dongjiao Minxiang, West Area, Beijing, 100730 People’s Republic of China

**Keywords:** Calcaneal fracture, Fracture maps, Fracture spectrograms, Fracture mapping, Fracture frequency spectrum

## Abstract

**Background:**

Calcaneal fractures are associated with numerous complications and a poor prognosis with significant long-term quality-of-life issues, regardless of treatment. Therefore, in-depth research into the underlying mechanism of calcaneal fracture is still of great interest, with the goal of improving treatment for patients suffering from this condition. This study aimed to investigate the relationship between the distribution of calcaneal fracture lines and their determinants, especially those related to the internal structure of the calcaneus. This goal was achieved by fracture maps created by copying and stacking fracture lines as viewed from six surfaces of the calcaneus.

**Methods:**

A total of 210 consecutive patients with 226 calcaneal fractures were retrospectively analyzed. Fracture lines were copied from a reduced 3D calcaneal fracture model and stacked on calcaneal templates to generate fracture maps. The stacked images of six calcaneus surfaces were also converted into spectrograms with MATLAB to highlight the fracture frequency at specific locations.

**Results:**

There were four concentrated bands of fracture lines and two fracture hot spots on the superior surface. Three dense bands of fractures were observed on the medial surface, and four fracture bands were observed lateral to the calcaneus. Vertical fracture lines dominated the anterior calcaneal fracture map. On the posterior surface, the fracture lines appeared to be centered superiorly. All fracture locations coincided with the interfaces between the trabecular groups.

**Conclusions:**

The fracture maps showed fracture patterns and recurrent fracture zones on all calcaneal surfaces. The shape of the talus and calcaneus and the architecture within the calcaneus, especially the arrangement of the trabeculae, are essential factors for calcaneal fractures.

**Supplementary Information:**

The online version contains supplementary material available at 10.1186/s13018-022-02930-y.

## Background

The calcaneus is the largest and most frequently fractured tarsal bone. Intraarticular fractures account for approximately 75% of calcaneal fractures [1].

Regardless of the treatment, calcaneal fractures are associated with numerous complications and poor prognosis with significant long-term quality-of-life issues. Outcomes in patients who have undergone surgery have been shown to be worse than outcomes in patients with other orthopedic conditions, but the reason remains unclear [1–3]. Therefore, in-depth research on the mechanism of calcaneal fracture is still of great interest for improving treatments.

The characteristics of calcaneal fracture fragments and lines are mainly determined by three aspects: the shapes and positions of the talus and calcaneus, the inner structure of the calcaneus, and the magnitude and duration of the force [4–7].

The relative position of the calcaneus to the talus determines the development of one of the two primary fracture lines [3, 7, 8]. The mechanical axes of the talus and calcaneus are exocentric. As axial loading is applied to the calcaneus through the talus, shear forces are directed through the posterior facet, resulting in the first sagittal primary fracture line. This fracture splits the calcaneus into the anteromedial fragment (sustentaculum fragment) and posterolateral fragment (tuberosity fragment). The fracture line may then continue anteriorly to break the calcaneocuboid facet.

The shape of the calcaneus and talus determines the formation of the initial fracture site and the second primary fracture. Previous research has explained the role of the wedge-like anterolateral process of the talus and the thick bony strut at the angle of Gissane in producing a typical original lesion [8–10]. Axial loading then drives the anterolateral process into the angle of Gissane in an ax-like manner, and the second primary fracture line appears, running medially and laterally. It may then continue to split the middle facet and anteromedial fragment [11].

In addition, specific fracture patterns also result from the internal architecture of the calcaneus, especially from the pattern of the trabeculae [1, 2, 8]. The authors observed a consistent area of sparse or absent trabeculae in the anterior portion of the calcaneus, which has been previously referred to as the neutral triangle or Ward’s triangle. The fracture patterns were visible in the calcaneus, beginning in the neutral triangle, where the primary fracture site and primary fracture line originate. Sabry and Athavale observed six different trabecular groups or patterns in the calcaneus and that there are weaker zones along the interconnections between the lamellae [2, 7]. The primary fracture pattern then proceeds according to one of the “paths of least resistance” along trabecular weaknesses, creating the secondary fracture.

The magnitude and duration of the force also contribute to fracture patterns. Mild trauma only results in primary fracture lines and the simple pattern of fracture mentioned above. As the force continues, the talus pushes the posterior facet and the underlying thalamic fragment into the body of the calcaneus. It also pushes the lateral wall outward and causes fracture and bulging of the lateral wall. Occasionally, the talus may press against the medial part of the calcaneus, especially when the calcaneus is varus, resulting in fractures of the sustentaculum or medial column of the calcaneus [1, 11].

Although there is much research on calcaneal fracture patterns and their influential factors [1–4], thus far, there has been little work demonstrating this relationship visually. With advances in computed tomography (CT) and 3D reconstruction software, a method known as fracture mapping has been developed to describe fracture lines in 3D [12–14]. Fracture mapping is utilized in this study to visually present the relationship between calcaneal fracture patterns and their influencing factors in an integrated manner.

## Methods

### Ethical approval

The study was conducted following the Declaration of Helsinki and was approved by the Ethics Committee of the Ethics Review Board of PLA General Hospital. Informed consent was obtained from all patients before their enrollment in this study.

### Patients

This study included 210 consecutive patients (226 calcaneal fractures) treated at the Department of Orthopedics, 8th Medical Center of PLA General Hospital between February 2017 and January 2021. The inclusion and exclusion criteria were as follows: inclusion criteria: (1) CT data for the calcaneus before treatment, (2) CT images with a thickness < 3.0 mm, and (3) age ≥ 18 years; exclusion criteria: (1) patients with pathological fractures, (2) patients with severe osteoporosis, systemic autoimmune diseases, diabetes or subtalar arthritis, (3) patients with calcaneal or lower extremity deformity before the fracture, (4) *patients* who had previously undergone foot or ankle surgery, and (5) patients with a healed fracture for which the fracture line could not be determined.

### Calcaneal templates

Essential Anatomy 3 (3D4 Medical, San Diego, CA, USA) was used to export the superior, inferior, anterior, posterior, medial, and lateral surface images of a 3D calcaneus positioned in the anatomic plane. The images were imported into Adobe Photoshop CC 2018 (Adobe, San Jose, CA, USA), and their transparency was adjusted to 50%; then, they were used as fracture mapping templates.

### Fracture mapping and fracture frequency spectrum

The DICOM file of calcaneal fracture CT data was imported into Mimics 20.0 (Materialise, Leuven, Belgium) software to construct a 3D model, and the calcaneus was then singled out. The split function of the software was used to identify and separate the fracture fragments, and the fragments were labeled with different colors. For patients with severe posterior articular surface displacement, movement and rotation were used to reduce fragmentation (Additional file 1). The position and direction of the whole calcaneus model were adjusted to be consistent with the templates of the six surfaces of the calcaneus as mentioned above, and a screenshot was then taken and saved. At the same time, the calcaneal fracture screenshot and template image were imported into Adobe Photoshop. After the fracture line was distinguished, a new layer was created, and the fracture line was traced with the pencil tool (the pixel size was 2, the opacity was 50%) (Additional file 2). The fracture line layer was saved as a separate image file. Finally, all the fracture line images for a given surface were superimposed with the stack function in Photoshop, and a gray map of the calcaneal fracture line was obtained after merging all the layers.

Fracture spectrograms were created based on the method of pixel superimposition. The pixel value of the fracture line was set to 1 pixel, all the fracture lines were superimposed on a grid, and the pixels at the same position were superimposed to obtain a grid composite image. Finally, the grid composite image was converted into a color image using two different color schemes. The first was the full spectrum color scheme, in which color was displayed by geometric interval. The class width was defined following geometric series, so the class width of each class was approximately equal, and the frequencies of observations were consistent. We used five groups to show the fracture line density at five levels. The second was the true color scheme: Color was displayed as a continuous variable; the more pixels that were superimposed, the brighter the color. Both schemes were implemented using MATLAB (MathWorks, Natick, MA, USA).

### Data analysis

The analysis of the fracture maps and spectrum was descriptive. Patient characteristics are summarized as the mean and standard deviation for continuous variables and as a percentage for categorical variables.

## Results

### Patient characteristics and fracture classification

A cohort of 210 patients (153 men, 57 women) and 223 fractures (131 right and 92 left) were included. The average patient age was 47.6 (range, 19–72) years. According to the Sanders classification, there were 15 type II, 16 type III, and 31 type IV fractures.

### Superior fracture map

**Fig. 1:**
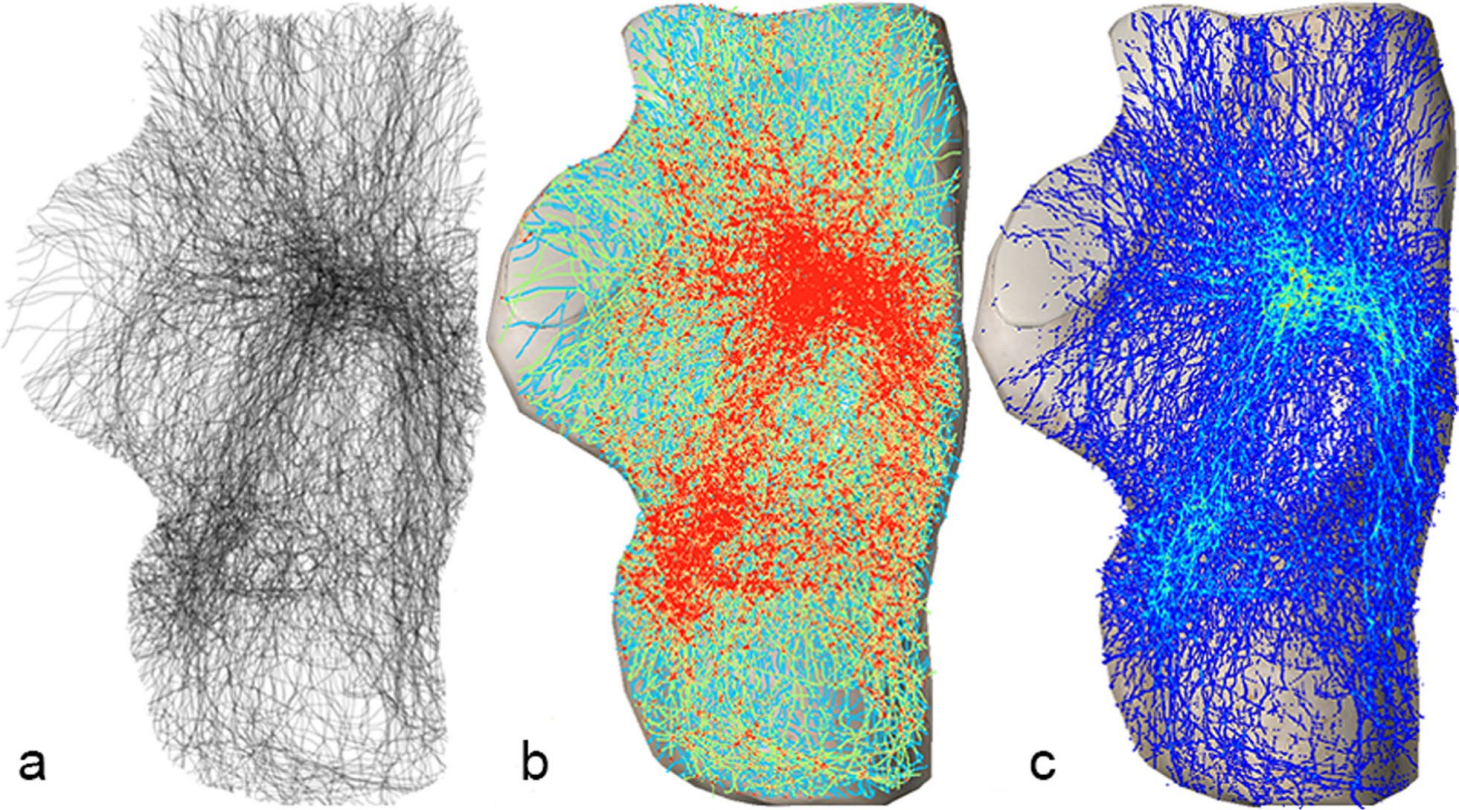
Superior fracture map: **a** Grayscale image, **b** Full spectrum, geometric interval image, **c** True color image. Two longitudinal and two transverse fracture bands can be observed in **a**, **b**. One anterior and one posterior fracture hot spot (the brightest green area) can be observed in **c**

From the superior fracture map, four fracture bands, in which the fracture lines were concentrated, and two hot spots, where the fracture lines intersected, could be distinguished (Fig. 1).

### Inferior fracture map

**Fig. 2:**
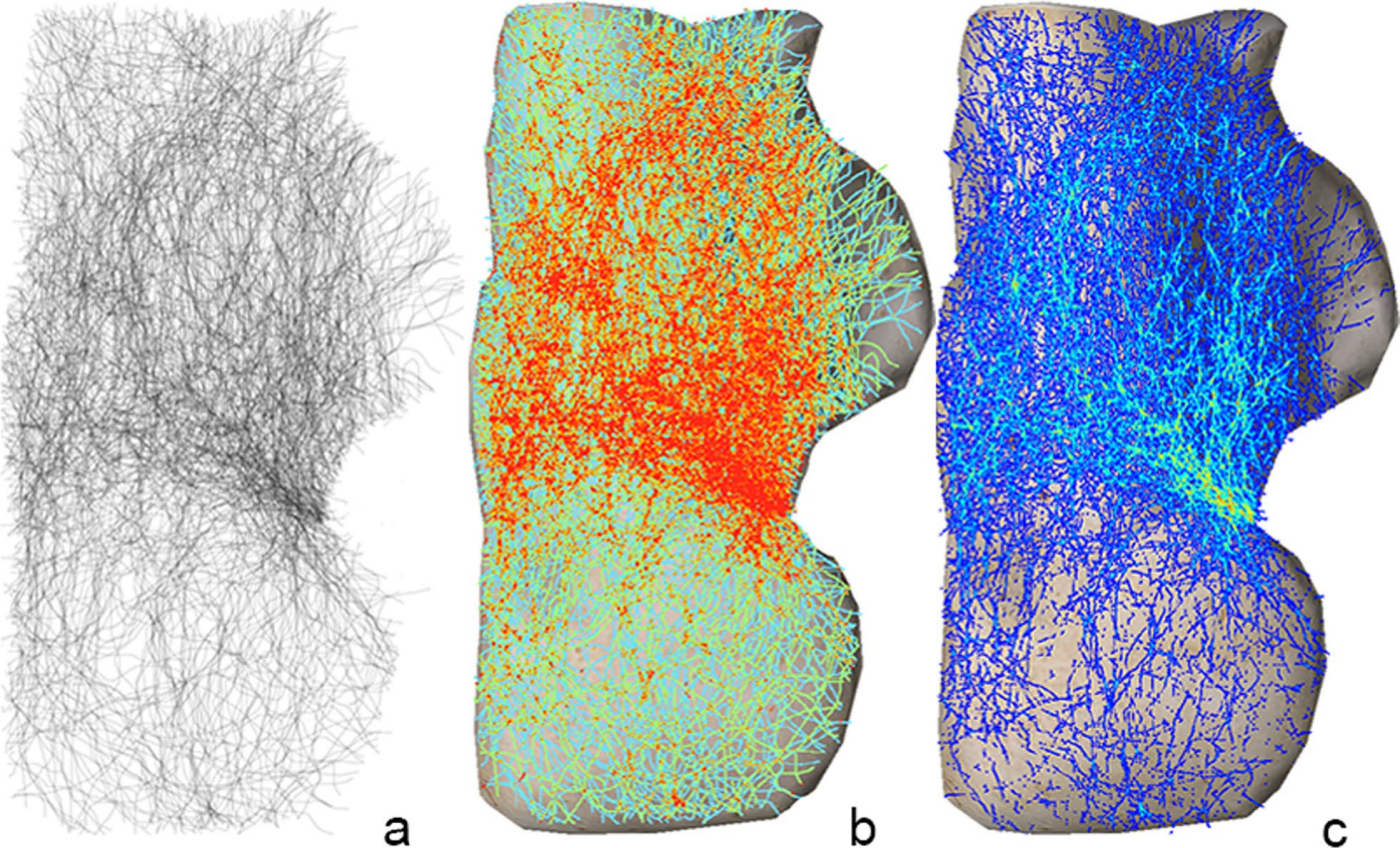
Inferior fracture map: **a** Grayscale image, **b** Full spectrum, geometric interval image: The red area indicates fractures concentrated in the middle anterior portion of the calcaneus. **c** True color image: The fracture line, originating from the posterior fracture hot spot (Fig. 1c), extends inferiorly

On the inferior map of the calcaneus, the fracture line is centered mainly on the middle part. Three areas had fewer fracture lines: the triangular region of the calcaneal head, the medial part of the sustentaculum tali, and the calcaneal tuberosity (Fig. 2).

### Medial fracture map

**Fig. 3:**
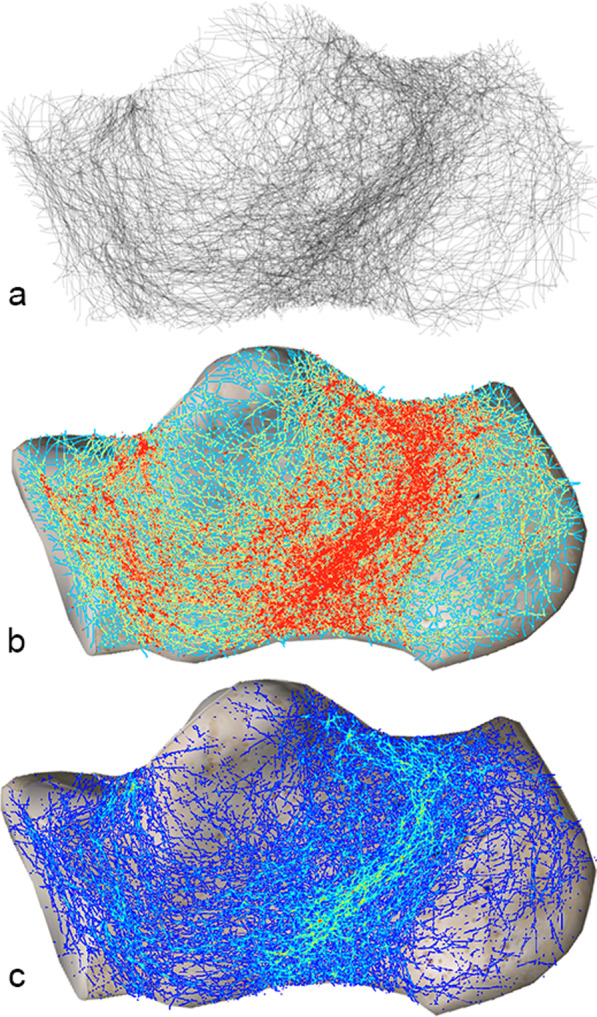
Medial fracture map. **a** Grayscale image, **b** Full spectrum, geometric interval image, **c** True color image. Three concentrated bands of fracture lines were observable

From the medial view, there were mainly three areas with dense fracture lines. Posteriorly to the subtalar facet, a band ran anteroinferiorly and terminated at the middle bottom of the body. Above this band, another high-density band ran horizontally from the posterior subtalar articular surface to the tuberosity. A longitudinal fracture zone was also observed in the calcaneocuboid area (Fig. 3).

### Lateral fracture map

**Fig. 4:**
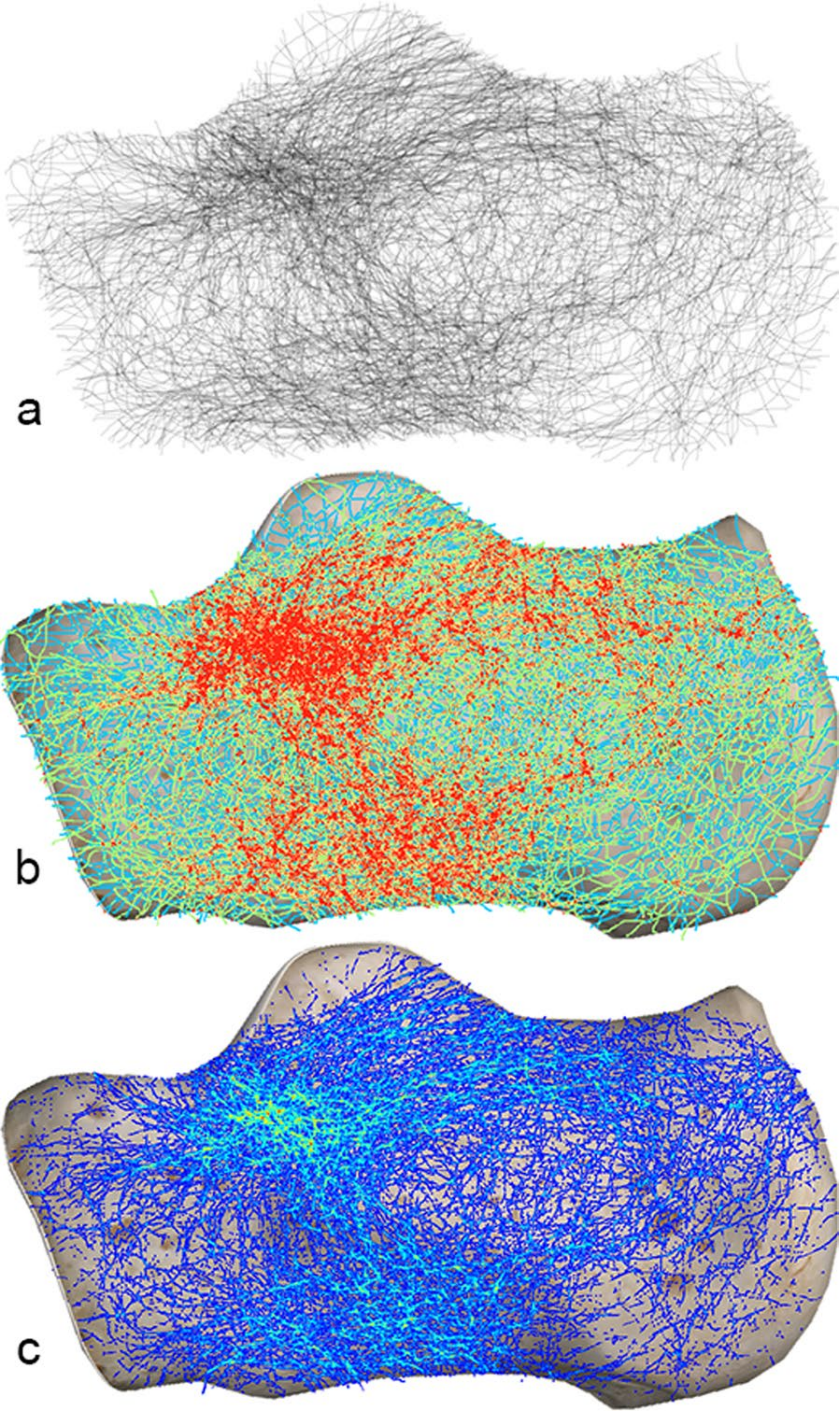
Lateral fracture map. **a** Grayscale image: At the superior part of the calcaneus, a horizontal band of fracture lines from the subtalar joint to the calcaneal tuberosity, extending anteriorly to the calcaneocuboid joint, was visible. **b** Full spectrum, geometric interval image. **c** True color image: Anterior fracture hot spots on the anterolateral aspect of the calcaneus (brightest area)

In the lateral view, the inverted “Y” fracture line area mentioned by Carr [4, 15] could be seen in the upper two-thirds of the calcaneus. However, a horizontal fracture area could also be seen in the lower part of the calcaneus, and the overall fracture pattern presents like a capital letter “H” (Fig. 4).

### Anterior fracture map

**Fig. 5:**
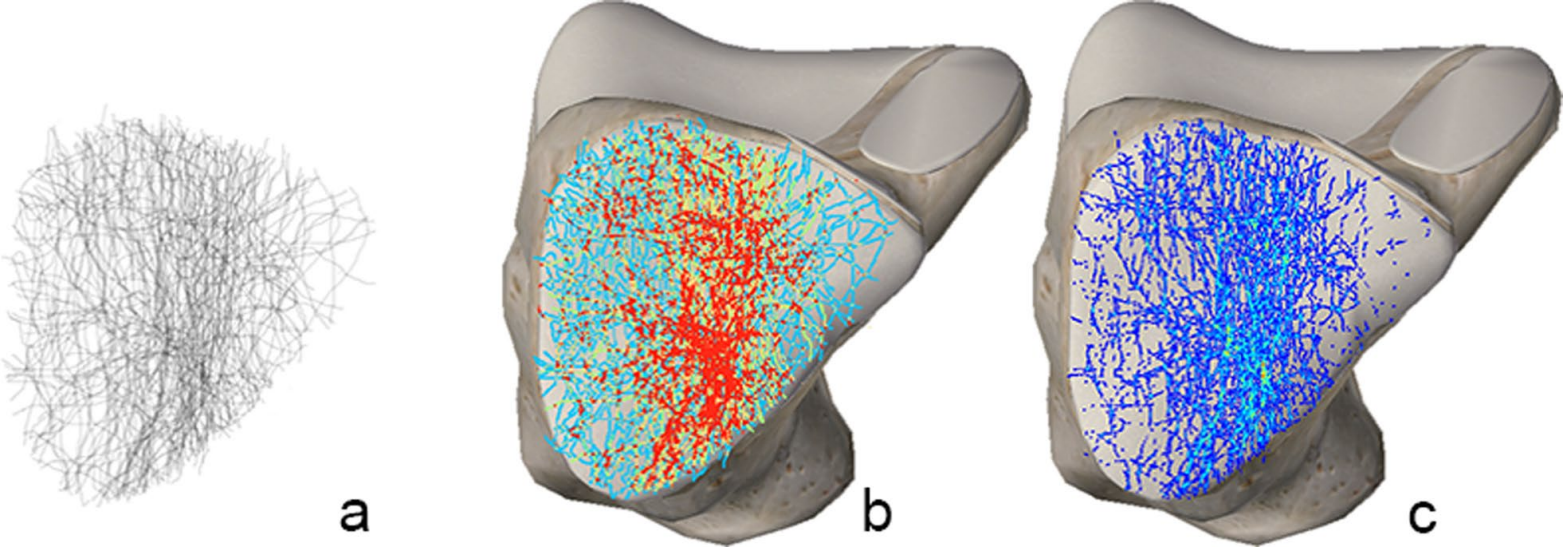
Anterior fracture map. **a** Grayscale image, **b** Full spectrum, geometric interval image, **c** True color image. Vertical fractures are dominant

Although the distribution pattern of the fracture lines is not obvious, the vertical fractures in the middle of the articular surface are still dominant (Fig. 5).

### Posterior fracture map

**Fig. 6:**
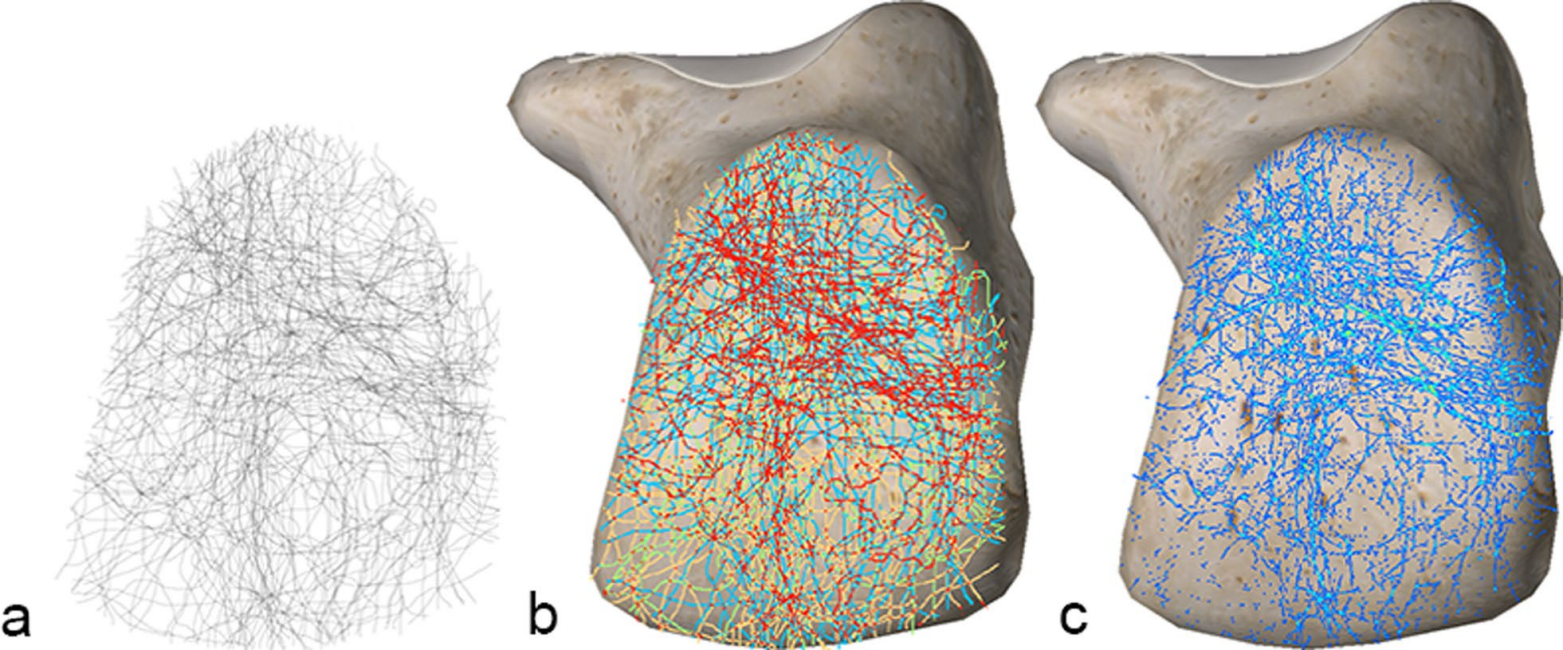
Posterior fracture map. **a** Grayscale image, **b** Full spectrum, geometric interval image, **c** True color image. The distribution pattern of the fracture lines is not obvious. However, it appears that there are more fractures in the upper region

We did not observe a regular fracture line distribution on the posterior side of the calcaneus. However, collectively, there were more fractures in the upper region than in the lower region (Fig. 6).

## Discussion

Calcaneal fractures are complex, and a regular fracture pattern is not as evident as at other sites. However, it is generally believed that the complexity of the fracture is mainly determined by three factors: 1. the shape and position of the talus and calcaneus; 2. the internal structure of the calcaneus, especially the distribution of the internal trabeculae; and 3. the magnitude of the trauma.

By copying and stacking fracture lines from the six surfaces of a 3D calcaneal fracture model, we obtained fracture line maps, which can visually show the relationship between the distribution of fracture lines and the determining factors.

**Fig. 7:**
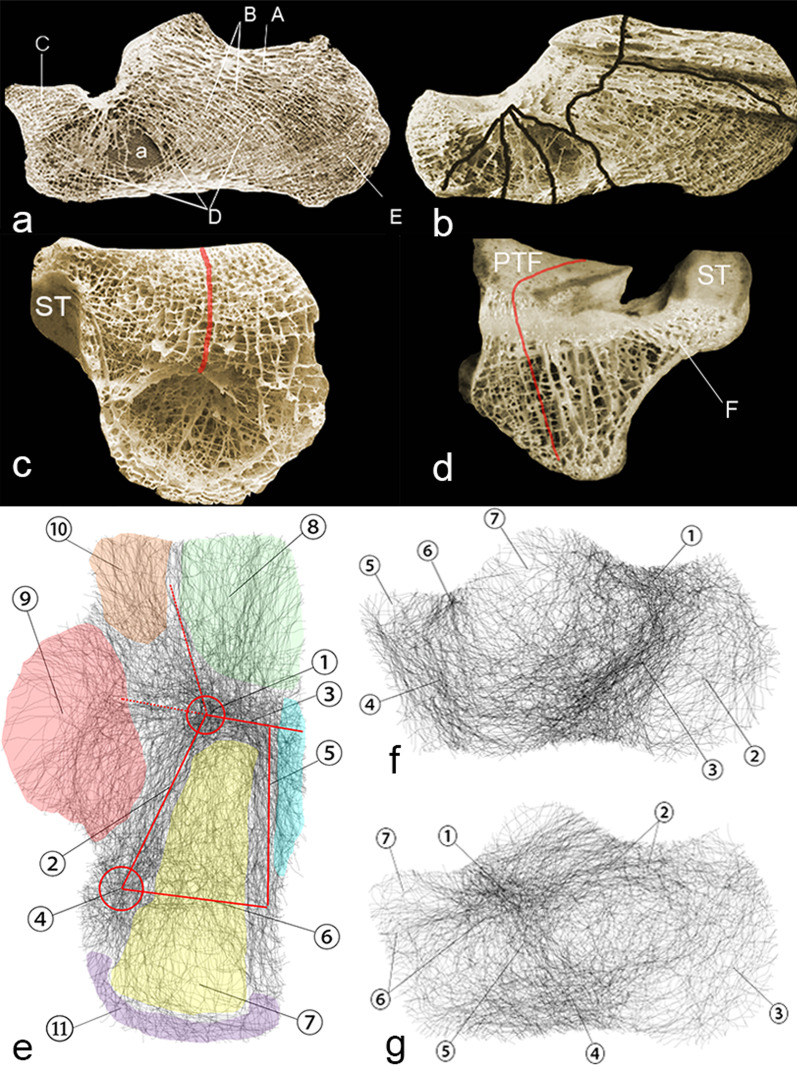
**a** Groups of trabeculae: A, B, C, D, E. Ward's triangle: a. **b** Interface of different groups of trabeculae. **c** Coronal section of the right calcaneus, viewed from the front, showing vertical lamellae and a thick transverse bony strut. **d** Coronal section of the calcaneus, viewed from behind, showing sagittal plates deep to the posterior talar facet and the horizontal cross bridges between them. The red arc marks the orientation of the interface between the lamellae, and this was also a site prone to fracture. (a), (b), (c), (d) Adapted from Ref. 8 with permission from Springer Nature. **e** Different trabeculae groups and fractures viewed from superior maps: ① Anterior fracture hot spot indicates the initial fracture position (anterior red cycle). ② Medial sagittal primary fractures, the red line marks the direction. ③ Transverse primary fractures; the red line marks the direction, and the red dotted line indicates its extension. ④ Posterior fracture hot spot: the position coincided with the posterior process of the talus. ⑤ Lateral secondary fracture lines. ⑥ Secondary transverse fracture lines. ⑦ Trabeculae group A. ⑧ The "pyramid" area of compact lamella. ⑨ Sustentaculum and medial wall. ⑩ Middle and anterior subtalar joint area. ⑪ Calcaneal tuberosities. **f** Medial fracture map. ① Posterosuperior horizontal fracture lines. ② Calcaneal tuberosity. ③ Fracture lines parallel to group D trabeculae. ④ Coronal fracture between the calcaneal neck and body. ⑤ Medial part of the compact pyramid. ⑥ Medial extension of the transverse primary fracture. ⑦ Sustentaculum tali and medial wall. **g** ① Anterior fracture hot spot indicates where the lateral process of the talus wedged. ② Posterosuperior horizontal fracture lines. ③ Calcaneal tuberosity. ④ Inferior horizontal fracture lines. ⑤ Vertical fracture passes through Ward’s triangle. ⑥ Anterior horizontal fracture lines. ⑦ Lateral part of a compact pyramid

The lateral process, shaped like a wedge, is the lowest part of the talus. When subjected to axial stress from the tibia, the lateral process will become wedged into the front of the Gissane angle like an ax, creating an initial fracture point (Additional file 3). A set of such points constitutes a hot spot region that can be observed on the superior fracture map (Figs. 1 and 7e-①). Then, originating from the initial fracture point, two primary fracture lines, the medial sagittal primary fracture line (Fig. 7e-②) and the anterior transverse primary fracture line (Fig. 7e-③), extend along the weakest interface. We can see that the dense bands of the two primary fracture lines converged at the anterior hot spot area (Fig. 7e-①). Immediately posterior to the subtalar facet, a transverse fracture strip severs the connection of the posterior subtalar articular facet and the calcaneal tuberosity (Figs. 1 and 7e-⑥), resulting in a compression fracture type.

Interestingly, the medial sagittal fracture line (Fig. 7e-②) converges with the posterior transverse fracture line (Fig. 7e-⑥) at another hot spot (Fig. 7e-④), as well as the anterior hot spot. The location of the posterior hot spot coincided with the site of the posterior process of the talus. In addition, the medial sagittal initial fracture line connects the anterior and posterior hot spots. We therefore hypothesized that the posterior process, similar to the anterolateral process, is the site of origin of the posterior initial fracture point when under axial stress (Additional file 3). The primary fracture line connecting the two points is the mechanically weakest area.

Detailed studies of the internal architecture and trabecular patterns of the calcaneus have been performed repeatedly [8, 16–18]. The cortical shell of the calcaneus is compact and thicker in the following areas: medial wall; inferior surface of the sustentaculum; articular areas; attachment area of the tendo calcaneus; medial and lateral tuberosities; and the area between the three plantar tubercles. In the calcaneus, six different groups of trabeculae can be observed [7, 8]:

Group A: This group is a compact set of trabecular rods extending from the upper part of the posterior subtalar facet to the upper one-third of the calcaneal tuberosity (Fig. 7a, b).

Group B: This group includes lamellae extending from the posterior subtalar facet to the medial and lateral tubercles (Fig. 7a, b). They are vertical, interconnected bony plates that have a sagittal orientation deep to the posterior talar facet. When traced posteroinferiorly, these plates break up into thin plates and branching rods, which diverge slightly from one another. They are also gradually twisted, assuming an arched course, and bridge the medial and lateral tubercles (Fig. 7c).

Group C: This set is an anterior extension from the lower edges of the sagittal lamellae of group B. These plates are stacked and run parallel to the anterior part of the superior surface of the calcaneus (Fig. 7a). Their lateral edges incline down sharply and become sagittal (Fig. 7b). This group appears like a pyramid, with the base at the cuboidal facet and apex at the thickened Gissane angle.

Group D: This group arises from the posterior surface of tuberosity in fine rods and extends anteroinferiorly. They intersect with the group B lamellae posteriorly, converge and condense in the form of horizontal plates inferiorly, parallel to the inferior surface of the calcaneus. Traced anteriorly, they again diverge in the form of rods, intersect group C lamellae, and extend up to the cuboidal articular facet (Fig. 7a).

Group E: This set comprises stacked plates running parallel to the posteroinferior surface of the calcaneal tuberosity and extends the area of attachment of the tendo calcaneus (Fig. 7a, b).

Group F: From the sustentaculum tali, this group of trabeculae runs downward and converges to join the compact and thickened medial wall of the calcaneus. They cross at right angles with lamellae that are directed horizontally (Fig. 7c, d).

In portions with thicker and compact cortical bone, few fractures occur. While stress perpendicular to that trabecular arrangement is tolerated well, the bone is biomechanically weakest parallel to these planes. Therefore, the fractures were usually concentrated near the interface of each trabecular group and lamellae (Fig. 7b, d and e).

From the superior map, we can see the sagittal primary fracture lines (Fig. 7e-②) located close to the interface between the medial part with compact cortical bone(Fig. 7e-⑨) and group A and B trabeculae (Fig. 7a and 7e-⑦). Inferior to the subtalar facet, the fracture dehisces along the vulnerable planes between the vertical and sagittal lamellae (Fig. 7c and d).

Transverse primary fractures (Fig. 7e-③) were situated between the Gissane angle and the pyramid-like group C trabeculae (Fig. 7a and 7e-⑧). The lateral secondary fractures (Fig. 7e-⑤) were sagittal and between the group A and lateral walls. Transverse secondary fractures (Fig. 7e-⑥) cut off the horizontal group A trabeculae and then formed a fracture parallel to the plane of group B (Fig. 7a and 7f-③).

From both the medial and lateral fracture maps, we can observe that there is a concentrated band of horizontally aligned fracture lines at the posterosuperior part of the calcaneus (Fig. 7f-① and g-②). These fractures may result from the separation of group A and group B trabeculae (Fig. 7a, b) [19]. From the lateral map, we can also see that the posterior horizontal lines extend anteriorly to the calcaneocuboid facet (Fig. 7g-②). These anterior horizontal fracture lines are the demarcation of the compact “pyramids” (Fig. 7a-c and 7e-⑧) and other trabeculae. The position of the longitudinal fracture line (Fig. 7g-⑤) was consistent with the area of sparse trabeculae called Ward’s triangle (Fig. 7a-a). At the inferior edge of the lateral surface, there is also a fracture concentration area (Fig. 7g-④). This area is where the lateral walls are separated from the base of the calcaneus. The superior horizontal fracture line, vertical fracture line, and inferior fracture line (Fig. 7g-②④⑤⑥) form a capital “H.”

## Conclusions

The findings of the present study, for the first time, vividly describe the relationship between the distribution of fracture lines and the internal architecture of the calcaneus. In addition, we verified that the shape of the talus and calcaneus, especially the lateral process of talus, plays a vital role in the initiation of fracture. In particular, we found a fracture hot spot on the superior calcaneal surface, and its location coincided with the site of the posterior process of the talus. So, we speculated that the posterior process of the talus also played an important role in the initiation of fracture.

This study has the following limitations: Some of the simulated fracture reduction will be affected by the image quality, thus affecting the position of the fracture line. When the image is matched with the template, a specific magnification change is needed, which may affect the position of the fracture line. Individual differences in the calcaneus can affect the outcome. However, in our opinion, the results are still credible, and our conclusions are in accordance with previous knowledge. These findings will be helpful for choosing treatments and improving internal fixation instruments.

## Supplementary Information

**Additional file 1.** Reduction of the fragments. **a** Before reduction, **b** After reduction.

**Additional file 2.** The method to copy fracture lines (Take the superior surface for example). The template was at the bottom, and the fracture image was on the template. A new layer was created on the top, and the fracture line was traced with the pencil tool of Photoshop.

**Additional file 3.** Anterior and posterior initial fracture point. The location of the anterior initial fracture spot coincided with the site of the lateral process of the talus (red cycle). The site of the posterior initial fracture spot was corresponding with the position of the posterior process of the talus (yellow cycle).

## Data Availability

The datasets used for analysis for the current study are available from the corresponding authors on reasonable request.

## Abbreviations

ST: Sustentaculum Tali
PTF: Posterior Talus Facet
