# Supplementary figures and images for: Calcaneal fracture maps and their determinants
Source: J Orthop Surg Res. 2022 Jan 21;17:39. doi: 10.1186/s13018-022-02930-y (PMC8780651; doi:10.1186/s13018-022-02930-y)

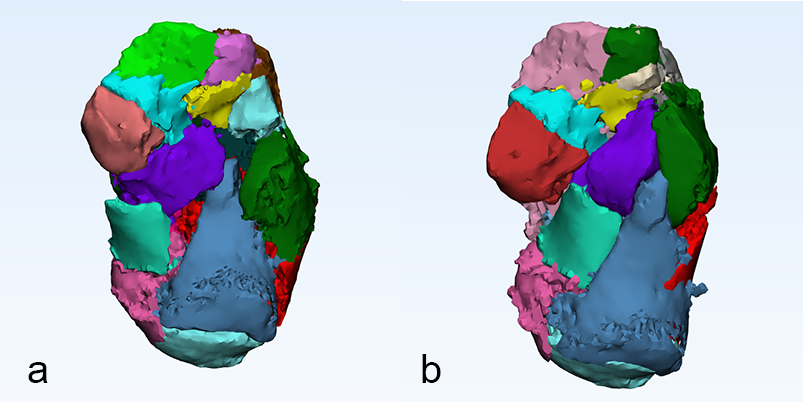


Additional file 1: Reduction of the fragments. **a** Before reduction, **b** After reduction.

Supplement: Supplementary file 1 — Additional file 1. Reduction of the fragments. a Before reduction, b After reduction. [file 13018_2022_2930_MOESM1_ESM.docx]
