# Supplementary material for: Calcaneal fracture maps and their determinants
Source: J Orthop Surg Res. 2022 Jan 21;17:39. doi: 10.1186/s13018-022-02930-y (PMC8780651; doi:10.1186/s13018-022-02930-y)
Supplement: Supplementary file 2 — Additional file 2. The method to copy fracture lines (Take the superior surface for example). The template was at the bottom, and the fracture image was on the template. A new layer was created on the top, and the fracture line was traced with the pencil tool of Photoshop. [file 13018_2022_2930_MOESM2_ESM.docx]

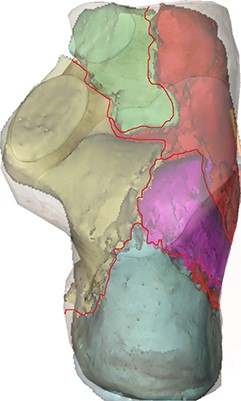


Additional file 2: The method to copy fracture lines （Take the superior surface for example）.The template was at the bottom, and the fracture image was on the template. A new layer was created on the top, and the fracture line was traced with the pencil tool of Photoshop.
