# Supplementary material for: Calcaneal fracture maps and their determinants
Source: J Orthop Surg Res. 2022 Jan 21;17:39. doi: 10.1186/s13018-022-02930-y (PMC8780651; doi:10.1186/s13018-022-02930-y)
Supplement: Supplementary file 3 — Additional file 3. Anterior and posterior initial fracture point. The location of the anterior initial fracture spot coincided with the site of the lateral process of the talus (red cycle). The site of the posterior initial fracture spot was corresponding with the position of the posterior process of the talus (yellow cycle). [file 13018_2022_2930_MOESM3_ESM.docx]

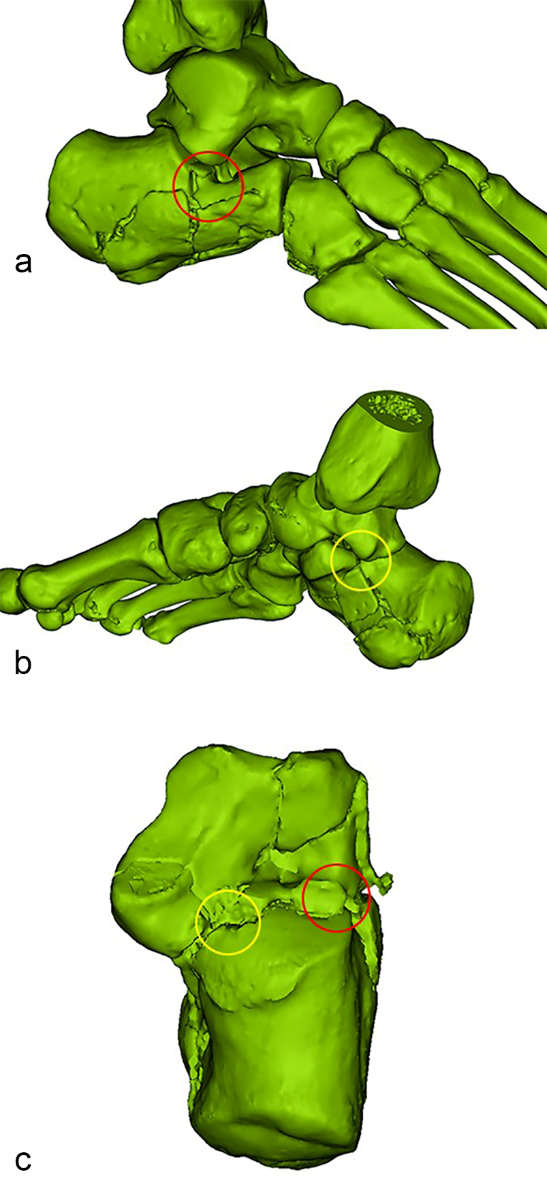


Additional file 3: Anterior and posterior initial fracture point. The location of the anterior initial fracture spot coincided with the site of the lateral process of the talus (red cycle). The site of the posterior initial fracture spot was corresponding with the position of the posterior process of the talus (yellow cycle).
